# Antibiotic resistance: it’s bad, but why isn’t it worse?

**DOI:** 10.1186/s12915-017-0423-1

**Published:** 2017-09-15

**Authors:** Nicholas Waglechner, Gerard D. Wright

**Affiliations:** Michael G. DeGroote Institute for Infectious Disease Research, Department of Biochemistry and Biomedical Sciences, McMaster University, 1280 Main Street West, Hamilton, ON L8N 4K1 Canada

## Abstract

Antibiotic natural products are ancient and so is resistance. Consequently, environmental bacteria harbor numerous and varied antibiotic resistance elements. Nevertheless, despite long histories of antibiotic production and exposure, environmental bacteria are not resistant to all known antibiotics. This means that there are barriers to the acquisition of a complete resistance armamentarium. The sources, distribution, and movement of resistance mechanisms in different microbes and bacterial populations are mosaic features that act as barriers to slow this movement, thus moderating the emergence of bacterial pan-resistance. This is highly relevant to understanding the emergence of resistance in pathogenic bacteria that can inform better antibiotic management practices and influence new drug discovery.

## The inevitability of resistance

**Fig. 1:**
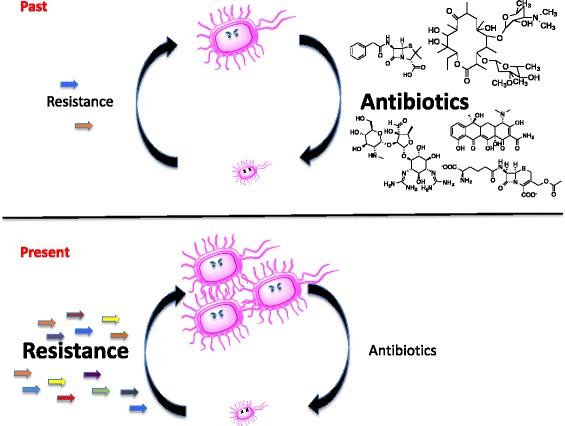
Past and present cycles of antibiotic discovery and resistance. For approximately 70 years (1930s–1990s) pathogenic bacteria and the diseases they cause were controlled with the discovery of many new antibiotic scaffolds and derivatives. Resistance inevitably emerged, by the capture of mobile resistance elements or intrinsic mechanisms, but was countered with new drug discovery. In the present situation, the lack of new antibiotic drugs and the rise of multi-drug-resistant pathogens that harbor many resistance elements presents a grave public health challenge

The history of antibiotic drugs over the past seven decades is one of cycles of discovery and clinical implementation, followed inevitably by resistance (Fig. 1). No classes of antibiotics are exceptions to this narrative. The evolution of resistance and its selection is, therefore, an intrinsic component of antibiotics, making them quite unique among drugs. While parallels exist in the anti-cancer field, the diversity of resistance mechanisms that bacteria deploy in response to antibiotics is unequaled and reflective of their long natural history. Given this experience, fair questions include what is the origin of this diversity, why is resistance apparently inevitable, and if it is, how is it disseminated among bacterial populations, what barriers (if any) prevent pan-resistance, and what are the ultimate prospects for the future of antibiotics?

## Antibiotics and resistance are ancient

The fossil record is consistent with the rise of single cell organisms approximately 3.5 billion years ago [1]. For the most part, these cells function through a common primary metabolism—carbon, nitrogen, and phosphorus acquisition and transformation into the elementary components of cells (amino acids, sugars, lipids), protein and nucleic acid synthesis, etc.—but they are differentiated by their relative abilities to generate secondary metabolites. These are the specialized molecules produced by microbes that are the result of natural selection within given environments and ecosystems. These metabolites include a broad diversity of compounds such as siderophores for metal acquisition, quorum sensing molecules deployed for inter-cell communication, and antibiotics, to name but a few. The latter have well-known antimicrobial activity but also at lower concentrations have pleiotropic effects on gene expression perhaps not related to their inhibitory phenotypes [2]. This activity profile that changes across a concentration gradient is a fascinating and poorly understood feature of antibiotics.

Molecular clock studies on the origins of the biochemical machinery required for antibiotic biosynthesis is consistent with their emergence as early as one billion years ago in the case of penicillins and resistance must be just as ancient [3, 4]. Direct evidence of ancient antibiotic resistance is, however, difficult to obtain. Ancient DNA studies conducted on preserved specimens hundreds to thousands of years old reveal that the portions of modern genomes that can be covered by ancient sequence reads have changed only little in comparison to modern counterparts [5–7]. Prospects for obtaining DNA from much older periods are poor given the challenge of preservation of biological samples. Microbial metagenome sequences obtained from permafrost have been collected and shown to possess comparable resistance to modern sequences [8]. For example, we reconstructed the complex vancomycin resistance cluster from metagenomic DNA isolated from 30,000-year-old Beringian permafrost. One of the resurrected enzymes demonstrated identical structure and function to modern mechanisms circulating in hospitals today [8]. Phylogenetic analyses also support the emergence of methicillin resistance in *Staphylococcus aureus* more than a decade before methicillin was synthesized and introduced in the clinic [9].

Another avenue to determine the frequency of antibiotic resistance in the environment is to survey isolated ecosystems, ideally where human influence has been minimal or non-existent. Such sites are challenging to identify in the Anthropocene but we have reported on genomic sequences obtained from bacteria sampled from a geologically isolated cave environment to reveal that organisms separated from phylogenetically close relatives for millions of years are resistant to multiple classes of antibiotics [10]. One hypothesis to account for this observation is that increased competition in nutrient-limited environments coupled with slow growth rates such as those found in sealed cave environments increases the value of antibiotic production and subsequently also resistance. Identifying antibiotic production in situ in these environments remains challenging. Such direct observations would illuminate the cycle of production and resistance and would clarify the role of antibiotics in such pristine natural environments. An alternative hypothesis is that the cadre of resistance elements that are common in various genera and species are only slowly lost, especially when located in the genome rather than mobile elements, even without selection and especially for slow growing microbes [11].

These lines of investigation all converge on the following: antibiotics and resistance are coupled, and both are ancient features of the prokaryotic lifestyle, deeply embedded in microbial genomes. Nevertheless, wherever the previous equilibrium between these phenomena had lain, humans have repurposed antibiotics for both medical and agricultural applications in modern times. Antibiotic production and use are now measured in tens of thousands of tons per year and, as a result, microbes are experiencing unprecedented levels of antimicrobial exposure. Consequently, we are experiencing a shift in the frequency of resistance in both environmental and clinical organisms in response to the anthropogenic use of antibiotics. Addressing this shift requires a detailed understanding of the strategies organisms use to become resistant to antibiotics and the forces that shape how they are employed and distributed.

## The evolution of resistance

Determinants of resistance can be classified into several categories depending on the nature of the antibiotic target and the biochemical mechanism of resistance [12]. The simplest case involves acquiring one or more mutations in the protein or gene target of the antibiotic that prevents binding, which is achievable through simple selective pressure and imperfect chromosome replication [13]. It is impossible to overcome or prevent this type of resistance from occurring as it reflects the intrinsic fidelity restrictions of DNA synthesis, and it is often the first outcome of antibiotic selection where a single gene modification can result in resistance. In cases where a host has multiple copies of an essential gene target, only one or a few of these copies may become resistant and a titration effect may be observed until enough resistant alleles are present to overcome the antibiotic. This can occur through successive acquisitions of mutations, through duplication of the target in the host genome, or by up-regulating the expression of the resistant target to titrate out the effect of the antibiotic [14].

There are also many ways to influence the effect of an antibiotic through the acquisition of genes that encode proteins that prevent or attenuate the effective binding of antibiotics to their molecular targets [15]. Similarly, there are enzymes that modify the antibiotic target to prevent drug binding [16]. In this case, acquisition of such elements means the host cell gains functionality de novo or acquires it from a pre-existing determinant from an external source. What governs the rate and propensity for generating entirely new resistance determinants is an open question.

Microbes have several ways of decreasing the effective concentration of an antibiotic. There are resistance determinants that can enzymatically act on an antibiotic to degrade or otherwise chemically modify (via donor molecules such as ATP or acetyl-CoA) an antibiotic such that it is no longer in an active form [17]. Examination of the structure and mechanism of such enzymes reveals that they are likely repurposed from catalysts with other functions in the cell, with perhaps weak activity towards the antibiotic that was enhanced through natural selection [18]. We coined the term ‘proto-resistance’ to refer to those genes and associated proteins, which are presumably performing other functions in their usual context but may be adapted by selection into sources of antibiotic resistance [12].

The intracellular concentration of antibiotics may be reduced through the action of efflux pumps, which are found ubiquitously in microorganisms [19]. Efflux pumps often have broad substrate specificities and can transport a wide variety of molecules across the cell membrane of the host, reflecting their primary roles in general detoxification. Narrow substrate range transporters are commonly found in natural product biosynthetic gene clusters and have specifically arisen to export products into the extracellular environment. These may represent a source of antibiotic resistance when found in a non-producing cell [20]. Another strategy to alter transport is to reduce cellular permeability to antibiotics. Gram-negative bacteria possess an outer membrane that greatly reduces the ability of many molecules to accumulate inside a cell at concentrations high enough to be inhibitory. Additionally, cells may evolve to be less permeable to antibiotics through selection on the number and expression levels of membrane-spanning porins that allow diffusion into the host [21]. It has been proposed that antibiotic selection was the driving force behind the evolution of the Gram-negative cell wall architecture [22].

When the antibiotic target is not a single gene product it is thought to be more difficult to evolve resistance. Examples of this include antibiotics that function by interacting with or disrupting the cell membrane itself, or antibiotics that target the precursors of cell structures like the building blocks of cell wall polymers [23]. In cases where resistance is known for such antibiotics, it is often achieved by accessing pre-existing diversity in these cell-wall structures. Altering the biosynthesis of these structures changes their physicochemical properties by using one or more alternative biosynthetic genes, and these changes can be leveraged to generate resistance [24].

Nearly all of these resistance mechanisms are observed in the biosynthetic gene clusters that encode the production of antibiotics. In some cases, the antibiotic producers’ own self-resistance genes have been argued to be the ancestors of the resistance determinants found in non-producing organisms [25]. It is unclear how or when this has occurred but it may have been recent in some cases [26]. Comparative studies using the increasing number of biosynthetic gene cluster sequences may help to establish a testable scenario for horizontal gene transfer to play a role in the mobilization of resistance determinants. Consider the birth of a novel biosynthetic cluster composed of components from pre-existing clusters that recombine to generate a new small molecule with antibiotic activity. The host carrying this biosynthetic gene cluster would require a form of self-resistance that leaves the new molecule intact lest the innovation is wasted while being able to exploit susceptibility in other organisms. Over time it may be expected that a copy of this self-resistance determinant, perhaps after a duplication event, would become part of the cluster as is frequently observed in other biosynthetic gene clusters and its expression become regulated with the cluster. Horizontal gene transfer (HGT) of entire clusters has been proposed to play a role in the evolution of secondary metabolism [27]. Once these genes become mobilized among phylogenetically related organisms, selection may favor HGT of the resistance genes alone among an even broader population to counter the advantage of antibiotic production.

## The spread of resistance through bacterial populations

**Fig. 2:**
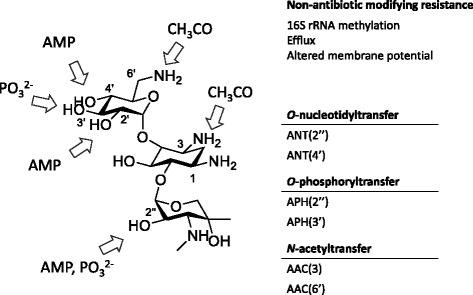
Gentamicin resistance, an example of genetic and biochemical diversity. Resistance to gentamicin occurs in a variety of ways. Altered membrane potential, efflux, and 16S rRNA methylation all confer resistance but leave the antibiotic unaltered. Various group-transfer reactions add phosphoryl, nucleotidyl, or acetyl groups at several positions on the molecule

While antibiotics and resistance are ancient, in most cases resistance to specific antibiotics has emerged independently several times. This is reflected in the diversity of resistance strategies that we currently encounter for all classes of antibiotics. The aminoglycoside antibiotics are a good example. Resistance can occur through efflux mechanisms, by target mutation to insensitive variants, by target modification, and by three distinct classes of chemical modification of the antibiotic molecule (Fig. 2). We can readily identify such resistance elements in many bacterial genomes of non-aminoglycoside producers and some have redundant mechanisms. How did this occur? One can imagine a scenario where producing organisms can establish an advantage in a local environment; resistance in a neighboring population (likely a producer of its own antibiotic) is either acquired via HGT or developed de novo, resulting in ecological ‘*détente*’ over time. The abundance of resistance elements in the chromosomes of environmental bacteria is consistent with such an idea, reflecting the long natural history of interactions among bacteria. Bacteria will, therefore, acquire, over time, resistance elements that reflect their current and past encounters with antibiotic producers. Unless the resistance element provides a significant fitness cost, such elements should persist in the chromosome unscathed, though perhaps transcriptionally attenuated. The number of ‘silent’ resistant elements in the chromosome of bacteria supports this hypothesis.

The situation in pathogens is different. Many pathogens are common, though minimal, components of the human microbiota or are specialists with narrow host ranges. By virtue of their unique environment (resource rich, shorter growth cycles, specialized ecological niches) they have developed fewer biosynthetic gene clusters encoding broad spectrum antibiotics, and have instead invested in molecular machinery to cloak themselves or otherwise evade the immune system. Given their ecological niche, they have limited the production of antimicrobial agents to highly specific and narrow spectrum agents such as bacteriocins. Consequently, they have fewer dedicated resistance elements in their chromosomes (the highly regulated AmpC in Enterobacteriaceae is the exception) and in general are highly susceptible to antibiotics. Historical collections of such organisms reveal a number of plasmids, but few mobile antibiotic resistance elements [28, 29]. With the dawn of the antibiotic era in the 1930s, this scenario has changed, and resistance genes have accumulated in these plasmids and often been mobilized to the chromosome.

A parsimonious explanation for this observation is that stochastic capture of resistance genes occurs on mobile elements regularly, but at an unknown frequency, in the environment (consistent with the accretion of resistance genes in environmental bacteria discussed above). The human use of antibiotics over the last seven decades has resulted in an intensive selective pressure that promotes fixation of mobile elements that carry resistance genes into pathogens. The initial capture of resistance determinants into mobile elements is, however, likely to be very rare. Given the genetic and mechanistic diversity of resistance in the environment, the frequency of such transfers must also be quite low, and obviously only occurs where such populations mix readily, such as in manures, water treatment facilities, and similar settings [30]. What is unknown is what makes one resistance element more likely to be mobilized over another. Again, the aminoglycosides provide a good example where new genes encoding antibiotic modifying enzymes appeared in pathogens regularly since their first clinical use in the late 1940s (kinases, acetyltransferases, nucleotidyltransferases), but ribosome methyltransferases, which confer high-level pan-aminoglycoside resistance in environmental bacteria, were not detected in pathogens until the early 2000s.

What this implies is that for any antibiotic used in medicine or agriculture, the spectrum of resistance elements in the environment must be cataloged and monitored for possible mobilization to the clinic. Such studies will provide an early warning system for the predictable development of resistance in pathogens. We expect, however, that it is unlikely that we can accurately predict which mechanisms will 1) escape the reservoir of resistance in the environment, and 2) be successfully mobilized across a broad spectrum of pathogens.

## Barriers to pan-resistance

If resistance is ancient, multifactorial, and mobile, why isn’t everything resistant to everything? Amid the alarm being raised about resistance, it is important to note that antimicrobial resistance is not total and universal. Even though certain important pathogens are increasingly resistant to some or all clinically used antibiotics, it should not be expected that all antibiotics everywhere will suddenly become useless against all bacteria. The bacterial resistome consists of all resistance determinants in prokaryotes [31]. In analogy to the pan-genome concept, it is difficult to say if the resistome is open (unlimited diversity) or closed (constrained diversity) and how much work is required to elaborate it fully. With informatic resources such as the Comprehensive Antibiotic Resistance Database (https://card.mcmaster.ca/), the in silico identification of resistance is advancing far ahead of the rate at which determinants are being biochemically characterized [32]. Nevertheless, questions may be asked about the structure of the resistome as it is currently understood and how it is changing as a result of the use of antibiotics. It has been argued that only the determinants identified in specific contexts need to be considered true resistance, compared to the operational definition that any determinant that causes a decrease in susceptibility is a resistance determinant [33]. The argument states that in order to properly estimate the risk of resistance the key event is that a determinant becomes decontextualized from its source, where it may not have functioned as a true resistance gene, and mobilized into a new context through HGT and only then it becomes a true resistance gene of public health or medical impact [33]. While we feel that this is an unnecessary restriction on the study of the resistome as a whole, the very reason for making this distinction illustrates a barrier to pan-resistance; many determinants of resistance, according to the operational definition, have not been observed outside of their original contexts, have not been mobilized into new hosts, and are not guaranteed to become mobilized in the future.

Fueled by low-cost genome and metagenome sequencing and growing databases that collect microbial genomic information, efforts have been made to describe the resistome in various genera. Our recent work in *Bacillus* and *Paenibacillus* has revealed that there are undiscovered determinants and mechanisms of resistance to be found [34]. Consistent with observations in the clinic, not all resistance determinants are found in all organisms. Why is this? What are the barriers to gene mobilization and capture? It has been suggested that phylogeny structures antibiotic resistance, where certain families of organisms are more likely to share more of their resistomes [11]. While this is broadly true at higher taxonomic levels, genomic surveys suggest the individual resistomes of different species are mosaic in nature [34]. There is certainly a role here as well for shared insertion sequences and other common elements that can favor homologous recombination and influence gene mosaicity.

## Humans and the bacterial resistome

Since antibiotics are excellent selective agents, it is critical to know how the actions of humans are affecting the bacterial resistome. Of the molecular mechanisms described above, selection for resistant mutations is the least avoidable. Ideal antibiotic targets are essential, and essential cellular components are highly conserved. Changes to one or more of these systems are often mildly or moderately deleterious. This is good news since it means that resistant organisms are often at a disadvantage in terms of absolute growth rate relative to sensitive organisms and that resistant variants of these targets may not easily substitute for the originals if acquired. This suggests the possibility that resistance can be reversed over time in the absence of selection, though in practice complete elimination from populations is unlikely [35].

Where resistance requires the presence of other determinants beyond the actual target of the antibiotic, many auxiliary factors come into play. These resistance determinants are either distributed narrowly within one or a few taxa, or they have become mobilized on genetic elements such as transposons, insertion sequences, plasmids, and phage and become subject to HGT. The prevailing view of bacterial genome dynamics suggests that HGT is constant and occurs more frequently between more closely related taxa. Efforts have been made to determine the general fitness cost of both passively carrying and actively expressing these determinants [36, 37]. The burden of carrying extra genetic material is thought to be low in general and expanding genome size does not appear to be a significant barrier to accumulating many resistance genes, at least in environmental bacteria. If average genome sizes of organisms are at equilibrium, there must also be a concomitant loss of genes [38]. A degree of plasticity seems to be an important facet of the evolution of bacterial genomes and suggests that in the absence of selection, antimicrobial resistance determinants along with many other genes may drift into and out of genomes with regularity [38]. This suggests that resistance may be reversed by drift. However, this requires sufficient time without selection, and it is unlikely that any lineage will remain unexposed to selection for long enough for all cells to be purged of resistance elements.

Whether or not mobilized resistance determinants become widely distributed depends on two factors in addition to selection by antibiotics: compatibility and proximity [39]. Compatibility refers to the ability of an organism to accept and express newly acquired genetic material. It may refer to the ability of the organism to participate in the exchange of DNA, but may also refer to the ability to express a functional resistance determinant in the correct context. Obvious barriers to compatibility can include plasmid compatibility mismatches, phage incompatibility, cell type incompatibility (a Gram-positive organism may not be able to express a Gram-negative outer membrane protein, for example), DNA GC content mismatch, codon bias, gene toxicity, and functional incompatibility where the recipient cell requires additional components not transferred from the donor [40]. All of these barriers seem to increase with increasing taxonomic distance. It has also been suggested that naturally competent organisms may be able to take up environmental DNA, which may include resistance determinants, and is subject to the same barriers. The types of antibiotic resistance determinants that are mobilized most easily might represent a class least affected by these barriers [41].

Though a variety of HGT mechanisms are capable of spreading resistance determinants, it remains unclear how recipient cells incorporate the proper regulatory control of newly acquired genes into their expression networks [42]. In open pan-genomes, the discovery of large and diverse accessory genomes in various bacterial families has spurred investigation into how and when these acquired genes may be expressed and has important consequences for our understanding of mobilized antibiotic resistance [43]. If the majority of these acquired genes are dormant, it may only require brief selection to activate their expression. Mobile elements can carry many different determinants and are participating in their own evolutionary dynamics. The capability to tune the expression of hitchhiking genes might help mobile elements mitigate deleterious effects of expressing genes in the wrong context, which would show that plasticity provides the raw material for selection [42].

The proximity of organisms refers to the contact that microbes have where they can exchange compatible genetic material. Organisms with narrow environmental niches, such as endosymbiotic bacteria or pathogens with restricted host ranges, may never have the opportunity to acquire foreign genetic material [44]. It would be an interesting exercise to determine if taxa with closed pan-genomes have similarly reduced resistomes and vice versa. Another example of a barrier refers to antibiotics with narrower spectrums. When an antibiotic only targets certain bacteria, for example, due to intrinsic resistance, there is no pressure on un-targeted bacteria to acquire resistance genes. Resistance to the glycopeptide antibiotic vancomycin is only observed in Gram-positive bacteria, despite the fact that Gram-negative bacteria may be susceptible to these antibiotics under certain circumstances, yet the resistance determinants have never been observed in Gram-negatives in spite of the fact that they co-exist in many environments with Gram-positives [45].

## Prospects—‘irresistible antibiotics?’

The evidence is now clear that the environmental resistome offers a near limitless diversity of antibiotic resistance elements that are at once highly specific—for example, inactivating enzymes—and broad—for example, efflux systems, to counter all antibiotics, even those synthesized in the lab. The ability to capture and mobilize genes horizontally through bacterial populations and to enhance these by natural selection from modest resistance activity into more robust phenotypes means that the development of an ‘irresistible antibiotic' is highly unlikely if not impossible. To suggest otherwise is to lack humility before the vastness of microbial genes and their history on the planet.

What is also clear is that we cannot completely avoid resistance. By understanding how resistance emerges and is spread through populations, we can better select and deploy the next generations of drugs [46]. This will require more understanding of the fundamental mechanisms of resistance and the precise degree to which HGT has shaped pathogen genomes in general and resistomes specifically. Fortunately, the unprecedented growth of sequence data is transforming the kinds of analysis that can be performed on whole prokaryotic genomes, of both clinically important organisms and the environmental organisms that surround us. This requires a concerted push for better surveillance, data sharing and dissemination, and the development of methods to leverage these data. Ultimately, this will be an effort of risk management that requires the participation of every stakeholder from policy makers down to basic researchers with clear communication to the public. Here we can benefit from the experiences from other industries, such as air transport, that has learned to identify potential risks and developed strategies to avoid them.
